# Influence of Sugar Headgroup on the Self-Assembly of Bioinspired Anionic Glycolipids

**DOI:** 10.1021/acs.langmuir.5c05353

**Published:** 2026-02-03

**Authors:** Giuliana Valentini, Álvaro Javier Patiño-Agudelo, Paulo Ricardo de Abreu Furtado Garcia, Watson Loh

**Affiliations:** Institute of Chemistry, 28132 State University of Campinas (UNICAMP), P.O. Box 6154, Campinas, SP 13083-970, Brazil

## Abstract

In this study, we investigated the micellization of bioinspired anionic glycolipids with distinct headgroups (xylose, rhamnose, and galactose) using surface tension, small-angle X-ray scattering (SAXS), and isothermal titration calorimetry (ITC) measurements across a range of temperatures. Surface and interfacial analyses revealed that their critical micelle concentration (CMC) is strongly influenced by the hydrophilicity of their sugar headgroups. SAXS data demonstrated that the aggregates are ellipsoidal micelles, with size and distortion influenced by their headgroup structure. Thermodynamic analyses indicate that micellization is driven by a delicate balance between enthalpic and entropic contributions, both being significantly affected by the sugar ring architecture, particularly the nature of the substituent at the C5 position. These findings show that the chemical features of the headgroup strongly influence the aggregate structure and micellization energetics of ionic glycolipids, providing relevant molecular-level insights for the rational design of new bioinspired amphiphiles.

## Graphical abstract

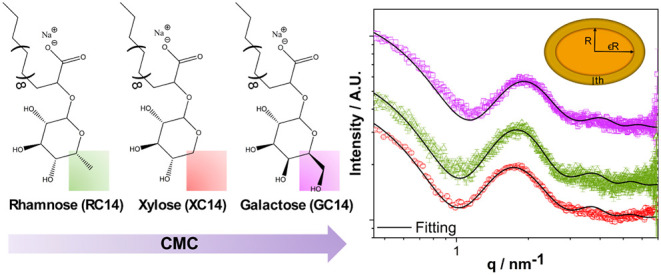

## 1 Introduction

Glycolipids are amphiphilic molecules consisting of a hydrophobic chain covalently bound to a hydrophilic sugar headgroup.^1^ This molecular architecture promotes their self-assembly in aqueous environments under defined conditions.^2–4^ They received significant attention because recent reports on bioinspired synthetic glycolipids produced through controlled carbohydrate synthesis^5^ show important applications in various fields, including environmental remediation and drug delivery.^6,7^ In addition, their ability to reduce surface and interfacial tensions, stabilize emulsions, and form supramolecular structures such as micelles and vesicles has attracted growing interest in colloid and interface science.^8–13^

Their physicochemical characteristics and structural diversity make glycolipids promising candidates for applications ranging from food to cosmetic or pharmaceutical formulations.^12,14–16^ Among the most common glycolipids, rhamnolipids, produced via microbial synthesis, have been studied, particularly for their surface activity and environmental compatibility.^16–19^ However, biosynthetic routes frequently yield complex mixtures of homologues, which complicates structural characterization and limits more systematic structure–property investigations.^20–22^

To overcome these challenges, synthetic bioinspired glycolipids have been used to provide molecularly well-defined systems.^9,23–26^ For example, Pacheco et al. 2017 ^22^ reported the synthesis of four diastereomers of the most common monorhamnolipid (α*-*rhamnopyranosyl*-*β*-*hydroxydecanoyl*-*β*-*hydroxydecanoate), enabling a systematic investigation of self-assembly mechanisms and of their surface and interfacial properties. Recently, Ma et al. (2025)^26^ synthesized a series of biomimetic glycolipids using an enzymatic approach motivated by their remarkable biological potential.^26^

The available literature demonstrates that the micellization behavior of natural nonionic glycolipids is highly sensitive to the molecular characteristics of the amphiphile,^27–30^ particularly regarding their critical micellar concentration (CMC) values. Conversely, the self-assembly properties of anionic glycolipids remain comparatively underexplored, and their thermodynamic profiles of aggregation are still scarcely characterized.^9,20^ Therefore, a key unresolved question concerns how structural changes in their sugar headgroup may modulate the surface activity parameters and, consequently, influence their aggregate structure and micellization thermodynamics.

In this work, we present a systematic investigation of the self-assembly behavior of bioinspired synthetic glycolipids. Specifically, we examine the aggregate structures formed by a series of sodium salts of α-glycosylated tetradecanoic acids (GC14, XC14, and RC14), in which galactose, xylose, or rhamnose is covalently attached to the α-carbon of the C14 fatty acid chain (Scheme 1). These systems were studied in phosphate buffer at pH 7.4, where the glycolipids are fully ionized, and at varying temperatures, using surface tension measurements to determine their interfacial parameters. Dynamic light scattering (DLS) and small-angle X-ray scattering (SAXS) measurements were employed to elucidate their micellar structures. To establish a correlation between the type of aggregate with the thermodynamic parameters governing self-assembly, isothermal titration calorimetry (ITC) experiments were conducted. The use of structurally well-defined glycolipids enabled precise analysis of how the sugar headgroup architecture influences micelle size, shape, and molecular packing, as well as their micellization energetics.

**Scheme 1:**
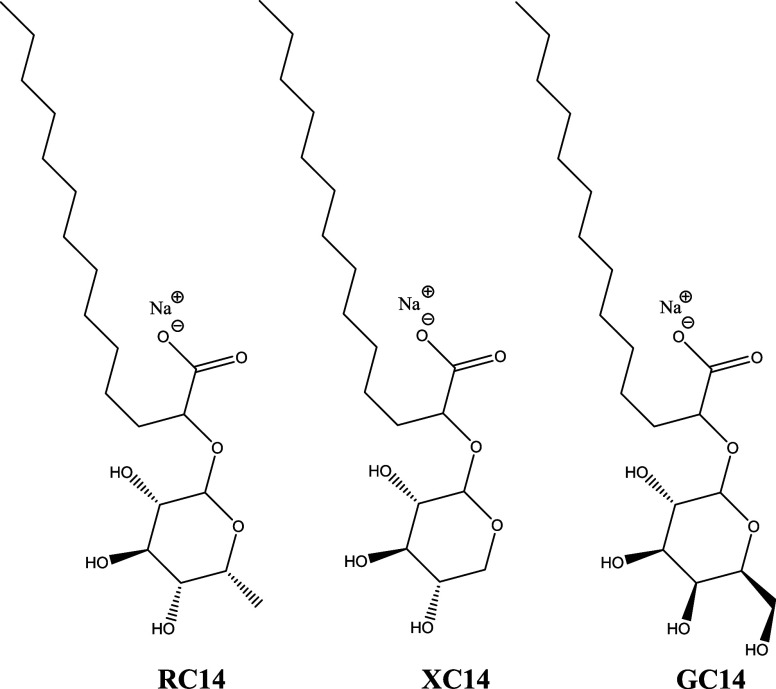
Chemical Structures of RC14, XC14 and GC14.

## 2 Materials and Methods

### 2.1 Materials and Preparation of Aqueous Solutions

Commercial sodium salts of α-glycosylated tetradecanoic glycolipids with three different sugar headgroups (galactose, xylose, and rhamnose) with purity >95% were purchased from Glycosurf (Salt Lake City, USA). Disodium hydrogen phosphate Na_2_HPO_4_ (99%) and sodium dihydrogen phosphate NaH_2_PO_4_ (99%) were purchased from Sigma-Aldrich (USA). All of these chemicals were used as received. Deionized water (resistivity <18 MΩ·cm at 25 °C) from a Milli-QII reverse osmosis system (Millipore, USA) was used to prepare all solutions.

A 100 mmol L^–1^ phosphate buffer at pH 7.4 was used as the solvent, with the pH adjusted using a calibrated pH meter, Titrino Plus, model 848, from Metrohm (Herisau, Switzerland). This buffer was selected to ensure complete ionization of the glycolipids, given the weakly acidic nature of its carboxylic group with their p*K*_a_ values lie between 4.0 and 4.3 (see Section S1 of the Supporting Information). The same buffer was used throughout the entire study.

### 2.2 Experimental Techniques

#### 2.2.1 Turbidimetric Determination of the Glycolipids Krafft Temperature

Measurements were conducted using a Shimadzu UV–vis spectrophotometer model UV-1900 (Kyoto, Japan) at 600 nm. The samples were gradually heated in a thermostatically controlled water bath under stirring, and the solution transmittance was monitored at regular temperature intervals after 5 min of equilibration, at a fixed glycolipid concentration of 90 mmol L^–1^ in phosphate buffer (pH 7.4). The Krafft temperature was determined as the point at which the transmittance reached its maximum and subsequently stabilized.^31^

#### 2.2.2 Interfacial Properties Obtained by Surface Tension Measurements

CMC and the interfacial molecular area (*a*_0_) of each glycolipid were determined by measuring their surface tensions as a function of their concentration, using an optical tensiometer Attension, Biolin Scientific (Göteborg, Sweden). All measurements were carried out at 45 °C, which corresponds to the minimum reliable temperature for comparing the glycolipids based on their Krafft points. The solutions droplet shape was analyzed using the instrument image analysis software, and surface tension (γ) values were calculated according to the modified Young–Laplace equation (Equation 1):^32^

1 $$\gamma =\frac{\Delta \rho \;g\;d^{2}}{\beta }$$

where Δρ is the density difference between the liquid and air, *g* is the gravity acceleration, *d* corresponds to the maximum diameter of the droplet, and β is a dimensionless parameter obtained by fitting the experimental profile to the theoretical model. The values of *a*_0_ were estimated from the surface tension curve below the CMC (Equation 2), using the Gibbs adsorption isotherm (Equation 3):^25,33^

2 $$a_{0}=\frac{1}{N_{a}\Gamma_{máx}}$$

3 $$\Gamma_{máx}=-\frac{1}{RT}\frac{d\gamma }{d\ln C}$$

where *N*_a_ is Avogadro’s number, (d*γ*/d ln *C)* represents the maximum slope, *T* is the experimental temperature, and *R* is the gas constant. The CMC value was determined by the intersection point of two lines obtained from the extrapolation of the linearly distinct regions of the surface tension versus glycolipids concentration curve.

#### 2.2.3 Micelles Structural Characterization Using Scattering Methods

##### 2.2.3.1 Dynamic Light Scattering (DLS)

The size of glycolipid aggregates were investigated by dynamic light scattering (DLS) using a CGS-3 goniometer system (ALV GmbH, Langen, Germany) operating in a pseudocross configuration at a 90° scattering angle. A 22 mW He–Ne laser (λ = 632.8 nm) provided the light source, and the intensity fluctuations were analyzed using an ALV 7004 multitau correlator. Glycolipid solutions based on sugar moieties were prepared at a concentration of 50 mmol L^–1^ in a buffer solution with pH 7.4. Toluene was employed to match the refractive index, and the experiments were conducted at a controlled temperature of 45 °C. The autocorrelation data were used to determine the translational diffusion coefficient, assuming a single particle size population. Under the premise that interparticle interactions were negligible, the hydrodynamic radius (*R*_H_) of the aggregates was determined using the Stokes–Einstein equation (Equation 4):^34^

4 $$R_{H}=\frac{k_{B}T}{6\pi \eta D}$$

where *k*_B_ is the Boltzmann constant, *T* is the experimental temperature, and η is the viscosity of the medium, assumed equal to that of the buffer solution. For aggregates exhibiting nonspherical shapes, *R*_H_ represents the radius of an equivalent hard sphere diffusing at the same rate, as previously reported in the literature.^32^

##### 2.2.3.2 Small-Angle X-ray Scattering (SAXS)

Small-angle X-ray scattering (SAXS) measurements were performed on a SAXSpoint 5.0 system (Anton Paar, Graz, Austria) equipped with a Primux 100 Cu microfocus source and an Eiger2 R 1 M detector (Dectris). For each sample, six data sets were recorded, each with a 30 min exposure, providing access to a scattering vector range of 0.2–7.0 nm^–1^. The scattering vector was defined as $q=\frac{4\pi \sin\theta }{\lambda }$ , where 2θ is the scattering angle and λ, the X-ray wavelength. All experiments were carried out at 45 °C.

Glycolipid solutions were transferred into quartz capillaries with an inner diameter of 1.5 mm and wall thickness of 0.05 mm. The capillaries were sealed using dental resin. Data processing, including azimuthal averaging and normalization, was performed using the SAXSanalysis software (Anton Paar). Scattering from dilute glycolipid solutions (0.5 mmol L^–1^, below their CMC) was measured under the same conditions and subtracted from the concentrated-sample profiles to remove background contributions.

The experimental data were fitted using a model of core–shell ellipsoids of revolution. The scattering intensity is expressed as Equation 5:^35^

5 $$I\left(q\right)=Sc\cdot P_{CS-Ell}\left(q,\rho ,R,\epsilon ,th\right)+B$$

In this formulation, *Sc* is the scale factor, ρ stands for the electron density contrast between the core and the surrounding shell, *R* designates the radius of the ellipsoid, ϵ defines its eccentricity, *th* refers to the thickness of the shell, and *B* represents the constant background contribution. The form factor associated with a core–shell ellipsoid of revolution is given by

6 $$P_{CS-Ell}\left(q,\rho ,R,\epsilon ,th\right)=\int_{0}^{\pi /2}F_{CS}^{2}\left(q,\rho ,R,\epsilon ,\alpha ,th\right)\sin\alpha \;d\alpha$$

7 $$F_{CS}^{2}\left(q,\rho ,R,\epsilon ,\alpha ,th\right)={\{V_{\mathrm{out}}F_{\mathrm{sph}}[q,r\left(R,\epsilon ,\alpha \right)]-\left(1-\rho \right)V_{\mathrm{in}}F_{\mathrm{sph}}[q,r\left(R+th,\epsilon ,\alpha \right)]\}}^{2}$$

8 $$F_{\mathrm{sph}}\left[q,r\left(R,\epsilon ,\alpha \right)\right]=3\frac{\sin\left[qr\left(R,\epsilon ,\alpha \right)\right]-qr\left(R,\epsilon ,\alpha \right)\cos\left[qr\left(R,\epsilon ,\alpha \right)\right]}{{[qr\left(R,\epsilon ,\alpha \right)]}^{3}}$$

where *r* (*R*, ϵ, α) = *R*(sin^2^α+ϵ^2^cos^2^α)^1/2^. In this context, *V*_in_ and *V*_out_ denote the volumes of the ellipsoidal core and of the complete particle (core plus shell), respectively, obtained from their geometrical semiaxes. The uncertainties reported for the fitting parameters correspond to the square roots of the diagonal elements of the covariance matrix obtained during the nonlinear least-squares minimization procedure. All modeling calculations were carried out with dedicated software implemented in C++.

#### 2.2.4 Determination of the Thermodynamic Parameters of Micellization

##### 2.2.4.1 Electrical Conductivity

Conductivity measurements were carried out using a 912 conductometer from Metrohm (Switzerland), previously calibrated with a 1 mmol L^–1^ KCl solution. For conductometric titration, a micropipette was used to sequentially perform 10 injections of 10 μL and 20 injections of 20 μL into 15 mL of pure water contained in a conductivity cell, while stirring at 400 rpm. The temperature was maintained at either 60 ± 1 °C (for all glycolipids) or 45 ± 1 °C (for RC14 and XC14 samples) using a water-jacketed cell connected to a thermostatic bath. A 1–3 min interval between injections was allowed to ensure the stability of the conductometric readings. Due to the high electrical conductivity of the phosphate buffer, conductivity measurements were performed in pure water, and details regarding the determination of the CMC and the micellar degree of dissociation (α) are provided in Section S2 of the Supporting Information.

##### 2.2.4.2 Isothermal Titration Calorimetry (ITC)

Isothermal Titration Calorimetry experiments were performed using a VP-ITC microcalorimeter (MicroCal Inc., USA). Stock solutions of the glycolipids were prepared in the same solvent (100 mmol L^–1^ phosphate buffer at pH 7.4) used to fill both the reference and sample cells, minimizing baseline thermal artifacts. Aliquots of 3–10 μL of the concentrated glycolipid solution were titrated into the reaction cell (cell volume of 1.44 mL) at regular intervals of 30–60 min, allowing sufficient time for baseline stabilization. The stirring speed was set to 600 rpm to ensure efficient mixing. Experiments were conducted at four temperatures: 45, 50, 55, and 60 °C. The lowest temperature was selected as the minimum reliable point for comparing the three glycolipids, considering their respective Krafft temperatures.

From the enthalpograms obtained by the ITC experiments, it was possible to determine the standard enthalpy change of micellization ( $\Delta H_{\mathrm{mic}}^{o})$ . The procedure to obtain this thermodynamic parameter consists of calculating the difference between two Δ*H*_obs_ values. The first $\left(\Delta H_{\mathrm{obs}}^{pre}\right)$ is obtained by fitting the observed enthalpy values in the premicellar region with the sigmoidal model, extrapolated up to the critical micelle concentration (CMC), identified as the inflection point of the enthalpogram. The second $\left(\Delta H_{\mathrm{obs}}^{\mathrm{post}}\right)$ is obtained from the same sigmoidal model in the postmicellar region, back to the CMC, as shown in Equation 9.^36,37^

9 $$\Delta H_{mic}^{o}=\Delta H_{\mathrm{obs}}^{\mathrm{post}}-\Delta H_{\mathrm{obs}}^{\mathrm{pre}}$$

To determine the change in Gibbs energy associated with micellization $\left(\Delta G_{mic}^{o}\right)$ , the values of CMC (in mol L^–1^) obtained by ITC were used. For anionic glycolipids, Equation 10 was applied:^27^

10 $$\Delta G_{\mathrm{mic}}^{o}=\left(2-\alpha \right)RT\ln CMC$$

where α is the degree of micellar dissociation obtained from electrical conductivity experiments. To complete the thermodynamic analysis, the standard entropy changes of micellization $\left(\Delta S_{\mathrm{mic}}^{o}\right)$ were determined using the fundamental Gibbs equation (Equation 11).

11 $$\Delta S_{\mathrm{mic}}^{o}=\frac{\Delta H_{mic}^{o}-\Delta G_{mic}^{o}}{T}$$

## 3 Results and Discussion

### 3.1 Krafft Temperature

To investigate the micellization of glycolipids, it is essential to determine first their Krafft temperatures, which define the minimum operational temperature required for the complete solubility of these anionic glycolipids in aqueous solution. This parameter is regulated by the surfactant chemical structure, commonly associated with their functional properties such as detergency, emulsification, solubilization capacity, and is strongly influenced by factors including salt concentration, pH, and solvent composition.^31,38–40^ Figure 1 shows the variation in transmittance as a function of temperature at a fixed glycolipid concentration of 90 mmol L^–1^ and pH 7.4.

**Figure 1:**
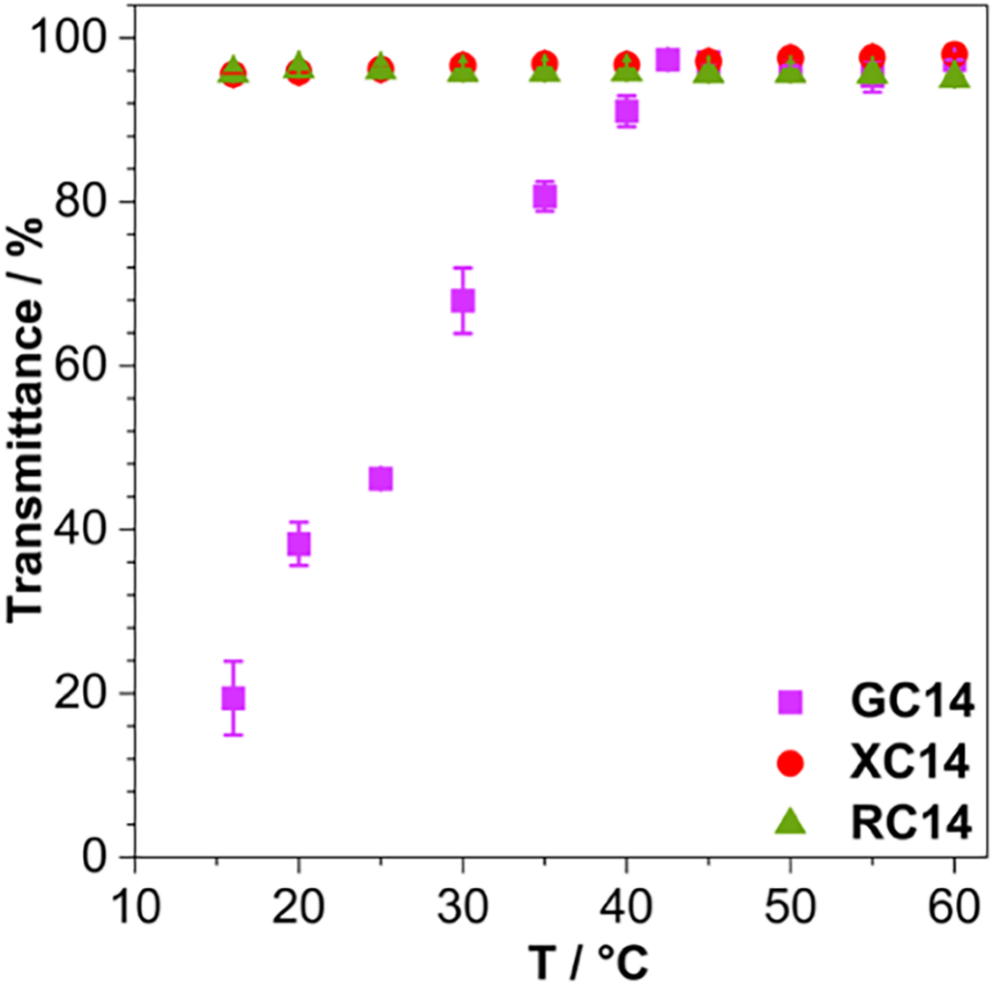
Temperature-dependent transmittance profiles of GC14, XC14, and RC14 at 90 mmol L^–1^ and pH 7.4.

Figure 1 illustrates the influence of the sugar headgroup on the glycolipid Krafft temperature. For GC14, the observed increase in transmittance with temperature reflects the solubilization of glycolipid crystals driven by thermal energy, reaching the Krafft temperature at 41 ± 1 °C. In contrast, XC14 and RC14 do not form crystals down to 16 °C under the same conditions, indicating significantly lower Krafft temperatures. These values are comparable to the range typically reported for other glycolipids.^28^ In an early investigation, we have determined Krafft temperatures for similar glycolipids in water, with much higher values, indicating that their complete ionization at the present pH of 7.4 favors their dissolution.^41^ Based on their Krafft temperatures, and despite the broad operational thermal range of XC14 and RC14, this study was restricted to 45–60 °C to enable consistent comparisons among the three glycolipids.

### 3.2 Surface and Interfacial Properties

As shown in Figure 2, the γ values decrease linearly with increasing glycolipid concentration, indicating the progressive adsorption of glycolipid monomers at the air–water interface. This decrease continues until the interface reaches energetic saturation, beyond which γ values remain constant despite further additions of glycolipid. The observation of similar minimum surface tension values for all glycolipids suggests that they display comparable interfacial activity. The concentration at which this plateau occurs defines their critical micelle concentration (CMC), and the corresponding values are listed in Table 1.

**Figure 2:**
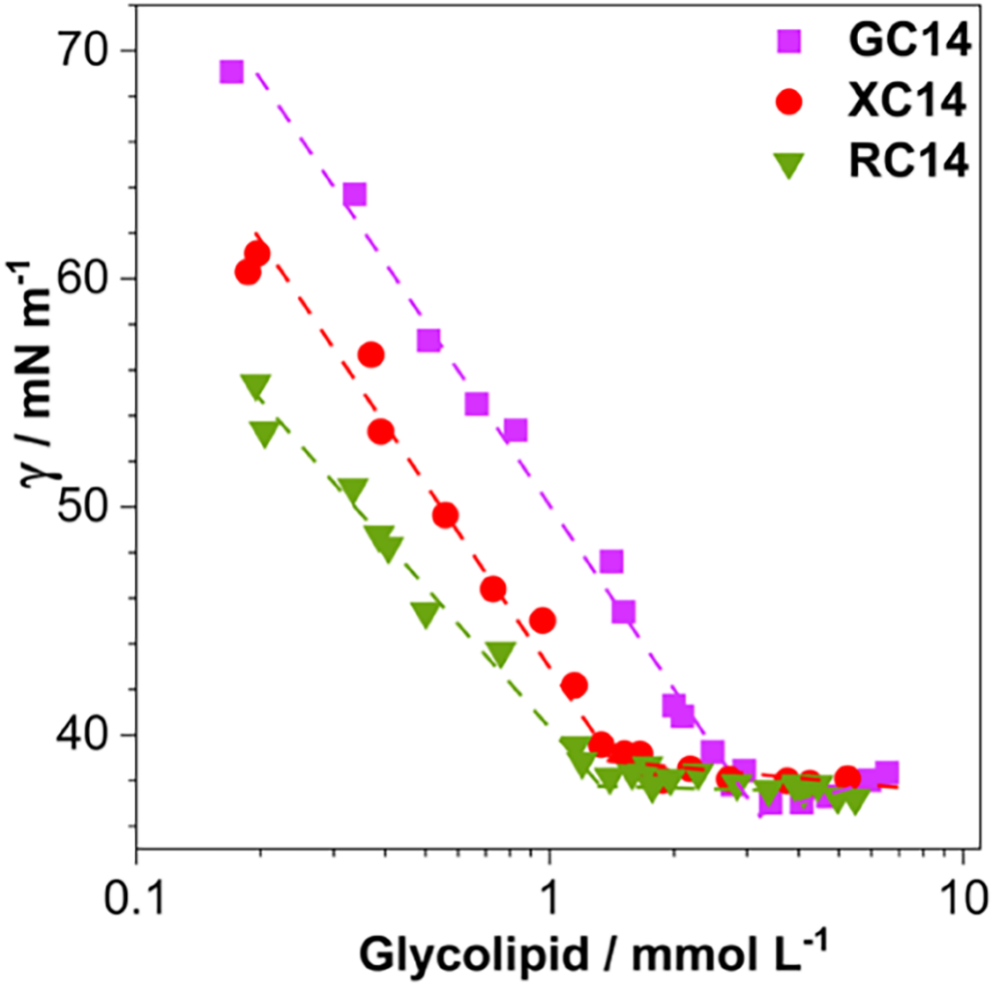
Surface tension values (γ) as a function of glycolipid concentration at pH 7.4 and 45 °C. The first point of the CG14 curve was excluded from the fitting due to its deviation from linear behavior, which is likely attributed to different interfacial packing.

**Table 1:** Interfacial Properties (CMC, γ_CMC_, and *a*_0_) for Glycolipids Obtained by Surface Tension Measurements at 45 °C and in Phosphate Buffer pH 7.4.

Glycolipid	CMC/mmol L^–1^	γ_CMC_/mN m^–1^	*a*_0_/Å^2^
GC14	3.3 ± 0.3	36.4 ± 0.2	42 ± 1
XC14	1.7 ± 0.1	38.1 ± 0.6	44 ± 1
RC14	1.3 ± 0.1	38.5 ± 0.3	52 ± 2

The CMC values exhibit a clear decreasing trend, from 3.3 ± 0.3 mmol L^–1^ for GC14 to 1.7 ± 0.1 mmol L^–1^ for XC14 and 1.3 ± 0.1 mmol L^–1^ for RC14. This trend correlates directly with the number of hydroxyl groups in the sugar headgroups (G > X = R): the fewer the hydroxyl groups, the lower the CMC value. The small difference between the CMC of RC14 and XC14 may be ascribed to the presence of a methyl group at C5 of the rhamno sugar ring, which renders the headgroup less hydrophilic than in XC14. Kegel et al. (2016)^21^ reported similar trends, showing that sugar solubility and CMC follow the same behavior.^21^ These findings underscore the possibility of modulating the micellization behavior of anionic glycolipids by tuning the solubility and polarity of their carbohydrate moiety. The presence of deprotonated carboxylic acid groups within their structures shifts the CMC to higher values compared to their nonionic analogs, despite the partial electrostatic shielding provided by the buffered medium.^36,42,43^

Another important parameter that can be derived from the surface tension results is the surfactant interfacial area, *a*_0_. As reported in Table 1, the observed trend in the *a*_0_ values is G < X < R, and reflects differences in the packing efficiency of the sugar headgroups at the air–water interface. This difference may arise from the simpler structure of the glycolipids investigated here, which contains only a single sugar headgroup and lacks bulky ester functionalities, resulting in less steric hindrance and enabling tighter packing.^20,44^ To assess how interfacial packing influences their micellar structures, a more detailed investigation was conducted, as described in the next section.

### 3.3 Structural Investigation of Glycolipid Aggregates by Scattering Methods

Dynamic light scattering (DLS) was employed to assess the size distribution of the aggregates, as inferred from their autocorrelation functions (see Section S3 of the Supporting Information). The autocorrelation curves for all samples display similar decay profiles, suggesting only one population of scatterers with comparable micelle sizes. The calculated *R*_H_ were 2.3 ± 0.1 nm, 3.0 ± 0.1 nm, and 2.5 ± 0.1 nm for GC14, XC14, and RC14, respectively, indicating that the absence of a substituent (−CH_3_ or −CH_2_OH) at C5 of the sugar ring leads to a larger aggregate, possibly due to higher aggregation numbers. These values suggest slightly larger micelles than predicted by the spherical approximation (see Section S4 of the Supporting Information) for C_14_ glycolipid micelles (radius ≈ 2 nm).^45^ The *R*_H_ values are also consistent with those previously reported for double short-chain rhamnolipids,^32^ suggesting a possible deviation from ideal spherical packing. To gain deeper insights into the structural organization of the micellar aggregates, SAXS experiments were subsequently performed.

SAXS is a suitable technique for investigating micellar structures, where differential scattering from the head groups enables the estimation of aggregate characteristics through model fitting. The scattering profile, as in Figure 3, characterized by the smoothed first minimum and irregular peak spacing, indicates anisotropy in particle shape.^46–48^ A satisfactory fit is achieved only with an ellipsoidal model (black line), which accounts for different principal axes and aligns with the internal distribution observed in the experimental data (colored points). All structural parameters obtained from the fitting of SAXS data are summarized in Table 2.

**Figure 3:**
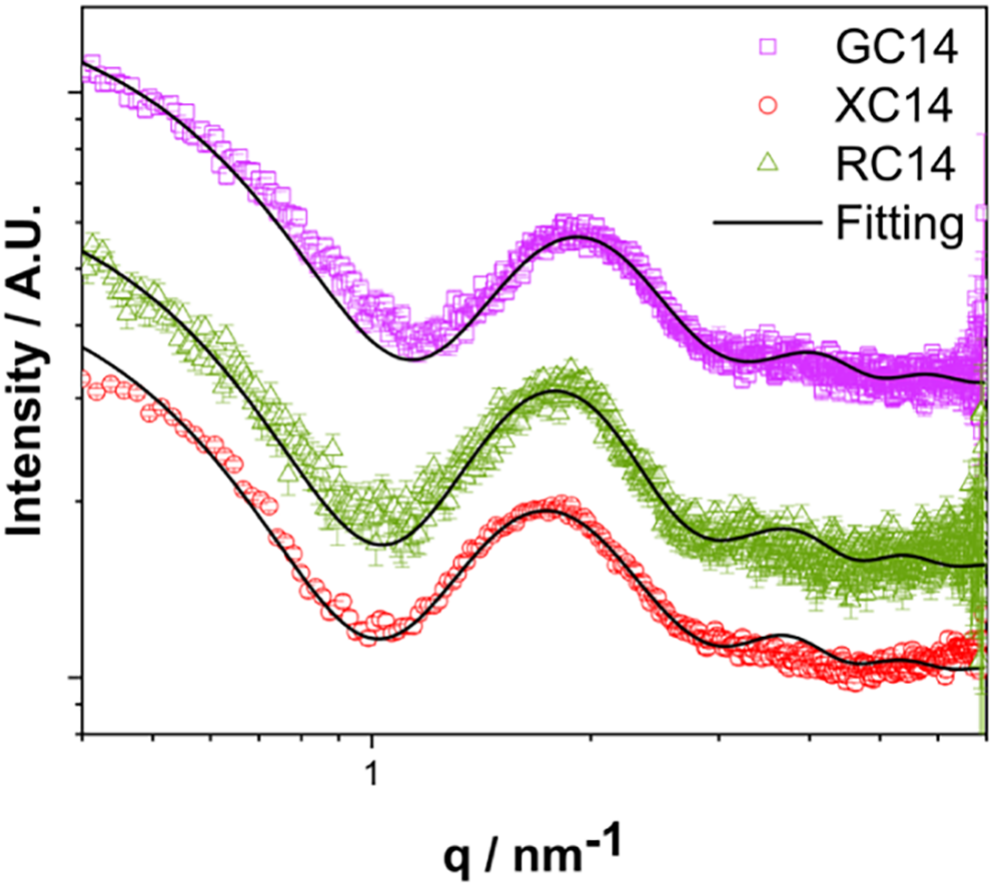
Representative small-angle X-ray scattering (SAXS) curves of GC14, XC14, and RC14 aggregates at 50 mmol L^–1^, pH 7.4, and 45 °C. The solid black lines correspond to the fitted curves using a core–shell ellipsoidal model.

**Table 2:** Aggregate Dimensions and Parameters Obtained from the Fitting of SAXS Curves for GC14, XC14, and RC14 Glycolipids at 50 mmol L^–1^, pH 7.4, and 45 °C.^a^

Parameters	GC14	XC14	RC14
***R***/nm	1.55 ± 0.01	1.68 ± 0.01	1.68 ± 0.01
***th*** **/nm**	0.44 ± 0.01	0.46 ± 0.01	0.45 ± 0.01
ϵ	1.51 ± 0.01	1.56 ± 0.02	1.47 ± 0.01
***V***_**core+shell**_ **/nm** ^**3**^	49.8	64.0	59.5
***N***_**agg core+shell**_	123	158	147
***V***_**core**_ **/nm** ^**3**^	23.5	31.0	29.2
***N***_**agg core**_	62	82	77
***N***_**agg area**_	174	198	152

a ***R*** represents the characteristic radius of the micelle; ***th*** corresponds to the shell thickness; **ϵ** corresponds to the eccentricity of the ellipsoid; ***V***_**core+shell**_ denotes the total micelle volume (core plus shell); ***N***_**agg core+shell**_ is the aggregation number calculated based on the total volume; ***V***_**core**_ is the volume of the hydrophobic core; ***N***_**agg core**_ is the aggregation number estimated using only the core volume; and ***N***_**agg area**_ is the aggregation number estimated using the molecular area obtained from surface tension measurements and the ellipsoidal dimensions determined by SAXS. The ***V***_**core+shell**_, ***N***_**agg core+shell**_, ***V***_**core**_, and ***N***_**agg core**_ were calculated described see the Section S4 of the Supporting Information.

The SAXS curves shown in Figure 3 exhibit profiles characteristic of globular micelles, all displaying a minimum in the *q*-value around 1 nm^–1^.^47–49^ However, the fitting results reveal small but structural differences among the aggregates in solution, as reflected by their parameters listed in Table 2. *R* values are very similar for XC14 and RC14, both at 1.68 ± 0.01 nm, indicating comparable dimensions for their micelle core, while GC14 forms smaller aggregates. In contrast, RC14 exhibits a slightly smaller eccentricity, ϵ = 1.47 ± 0.01 nm, suggesting greater compaction resulting in a less distorted ellipsoidal micelle. *th* values, that may be associated with the polar sugar headgroups, remained essentially constant for all the three systems (GC14: 0.44 ± 0.01 nm; XC14: 0.46 ± 0.01 nm; RC14: 0.44 ± 0.01 nm). This implies no significant differences in structural integrity or sugar headgroup packing within the micelle shell, based on the model used to account for these SAXS data.

Also, regarding their micelle size, Table 2 compares aggregation numbers estimated considering only the core volume (*V*_core_) and, additionally, including the core + shell volume (*V*_core+shell_). A difference can be seen between the two aggregation number estimations. Regarding the *N*_agg core_ estimates, these values approximate the aggregation number of a C_14_ perfect sphere estimated using a geometrical approximation (*N*_agg_ around 73).^45^ Considering the aggregate shell, the aggregation numbers (*N*_agg core+shell_) assume values of 147, 123, and 158 for RC14, GC14, and XC14, respectively. While the former values are likely to be underestimates for not considering the contribution from headgroups, the latter are probably overestimated because they should include water the volumetric contributions from water solvation molecules. The aggregation numbers estimated from the surface area (*N*_agg area_) for GC14 (174), XC14 (198), and RC14 (152) are consistently larger than those obtained from the total particle volume (*N*_agg core+shell_). The resulting *N*_agg area_/*N*_agg core+shell_ ratios (1.41, 1.25, and 1.03) combined with a shell thickness (∼0.45 nm) that aligns closely with sugar ring dimensions (∼0.5 nm) confirm that the *V*_core+shell_ model provides a more physically consistent description of a compact, moderately hydrated headgroup layer. However, these differences are not captured by the DLS-derived *R*_H_ values.

The smaller size and smaller *N*_agg core+shell_ observed for GC14 and RC14, when compared with XC14, can be ascribed to specific headgroup interactions, considering that the hydrophobic chain is identical among the three glycolipids. Comparing the three glycolipid monomers, their CMC values suggest that rhamnose has the least hydrophilic glycolipid (smallest CMC), while galactose appears as the more hydrophilic one, possibly arising from interactions related to their different C5 substituents on the sugar moiety. In particular, the −CH_3_ and −CH_2_OH groups are likely to promote more effective dispersive interactions, an effect not attainable with XC14. As for their micelle size, it is interesting that XC14, the glycolipid with the smallest C5 substituent (−H), forms the largest aggregates. The observed variations in shape anisotropy and shell thickness reveal how headgroups dictate molecular packing. These findings stress that varied interactions, including sugar headgroup hydration and, importantly, sugar–sugar interactions, contribute to determining the aggregate size.

Recently,^41^ we characterized similar systems in a concentrated glycolipid regime in water, where the form factor fitting used to account for those SAXS data remained consistent with prolate shapes, with dimensions comparable to those described in the present study. This indicates that micelle dimensions are quite similar in diluted phosphate buffer and aqueous environments, despite the former representing fully ionized surfactants.^41^ In addition, a similar core–shell ellipsoidal model was recently applied to describe related systems,^47,48^ where the distinction between core and shell strongly depends on certain predefined parameters and the implementation of the core–shell interface. In previous studies on systems containing surfactin^47^ and other glycolipids,^48^ the *th* values were modeled to extend up to, or even beyond, the length of the hydrophobic chain, indicating that the values of the hydrophilic shell strongly depend on the model chosen for data analyses.^47,48^

### 3.4 Micellization Thermodynamics and the Role of Sugar Headgroup

As discussed in previous sections, the nature of the headgroup in glycolipids significantly influences their interfacial parameters and the structure of their aggregates. To elucidate the molecular energetic implications of these differences, we conducted a thermodynamic study of their aggregation using ITC measurements at varied temperatures. Representative thermograms are presented in Figure 4, and all calculated thermodynamic parameters are summarized in Table 3 and Figure 5 (individual ITC curves are also presented in Section S5 of the Supporting Information).

**Figure 4:**
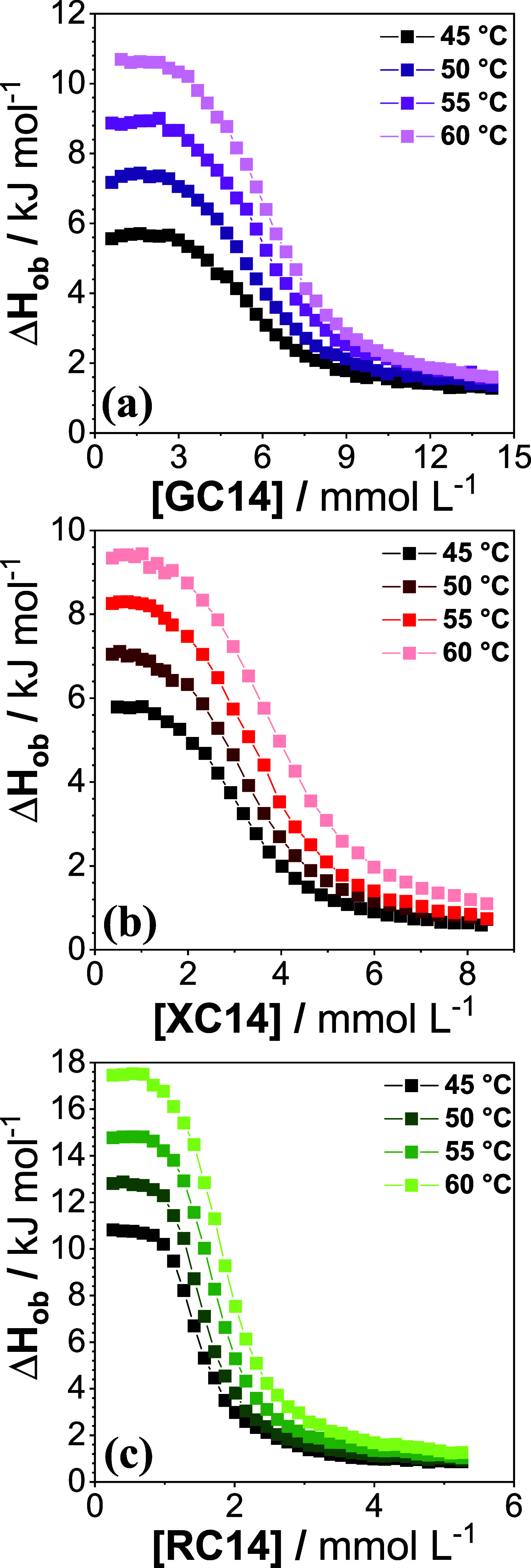
ITC curves for the micellization of (a) GC14, (b) XC14, and (c) RC14 measured in phosphate buffer, pH 7.4, at 45, 50, 55, and 60 °C. The continuous lines are guides for the eyes.

**Table 3:** Degree of Ionization (α), Critical Micelle Concentration (CMC), Enthalpy Change of Micellization $\left(\Delta H_{mic}^{o}\right)$ , Gibbs Free Energy Change of Micellization $\left(\Delta G_{\mathrm{mic}}^{o}\right)$ , and Entropy Change of Micellization $\left(T\Delta S_{\mathrm{mic}}^{o}\right)$ for GC14, XC14, and RC14 in Phosphate Buffer (pH 7.4).^b^

	*T/*°C	α^a^	CMC/mmol L^–1^	$\Delta H_{\mathrm{mic}}^{o}$ /kJmol L^–1^	$\Delta G_{\mathrm{mic}}^{o}$ /kJmol L^–1^	$T\Delta S_{\mathrm{mic}}^{o}$ /kJmol L^–1^
**GC14**	45	0.24 ± 0.07	5.63 ± 0.05	–4.45 ± 0.03	–24 ±1	20 ± 1
	50		5.71 ± 0.05	–6.05 ± 0.03	–24 ± 1	18 ± 1
	55		6.01 ± 0.04	–7.41 ± 0.07	–25 ± 1	17 ± 1
	60		6.19 ± 0.04	–9.47 ± 0.09	–25 ± 1	15 ± 1
**XC14**	45	0.45 ± 0.04	3.13 ± 0.03	–5.39 ± 0.06	–23.6 ± 0.6	18.3 ± 0.6
	50		3.25 ± 0.03	–6.48 ± 0.06	–23.9 ± 0.6	17.4 ± 0.6
	55		3.45 ± 0.02	–7.76 ± 0.07	–24.0 ± 0.6	16.2 ± 0.6
	60		3.79 ± 0.02	–8.51 ± 0.06	–23.9 ± 0.6	15.4 ± 0.6
**RC14**	45	0.22 ± 0.01	1.51 ± 0.02	–10.25 ± 0.07	–30.4 ± 0.2	20.2 ± 0.2
	50		1.58 ± 0.01	–12.09 ± 0.07	–30.7 ± 0.2	18.6 ± 0.2
	55		1.73 ± 0.01	–13.84 ± 0.07	–30.7 ± 0.2	16.9 ± 0.2
	60		1.84 ± 0.01	–16.34 ± 0.09	–30.9 ± 0.2	14.5 ± 0.2

a These values were measured in water (see text and Supporting Information).

b The errors for $\Delta G_{mic}^{o}$ and $T\Delta S_{mic}^{o}$ were calculated by error propagation. The CMC values reported by ITC follow the same trend observed in the surface tension data at a temperature of 45 °C.

**Figure 5:**
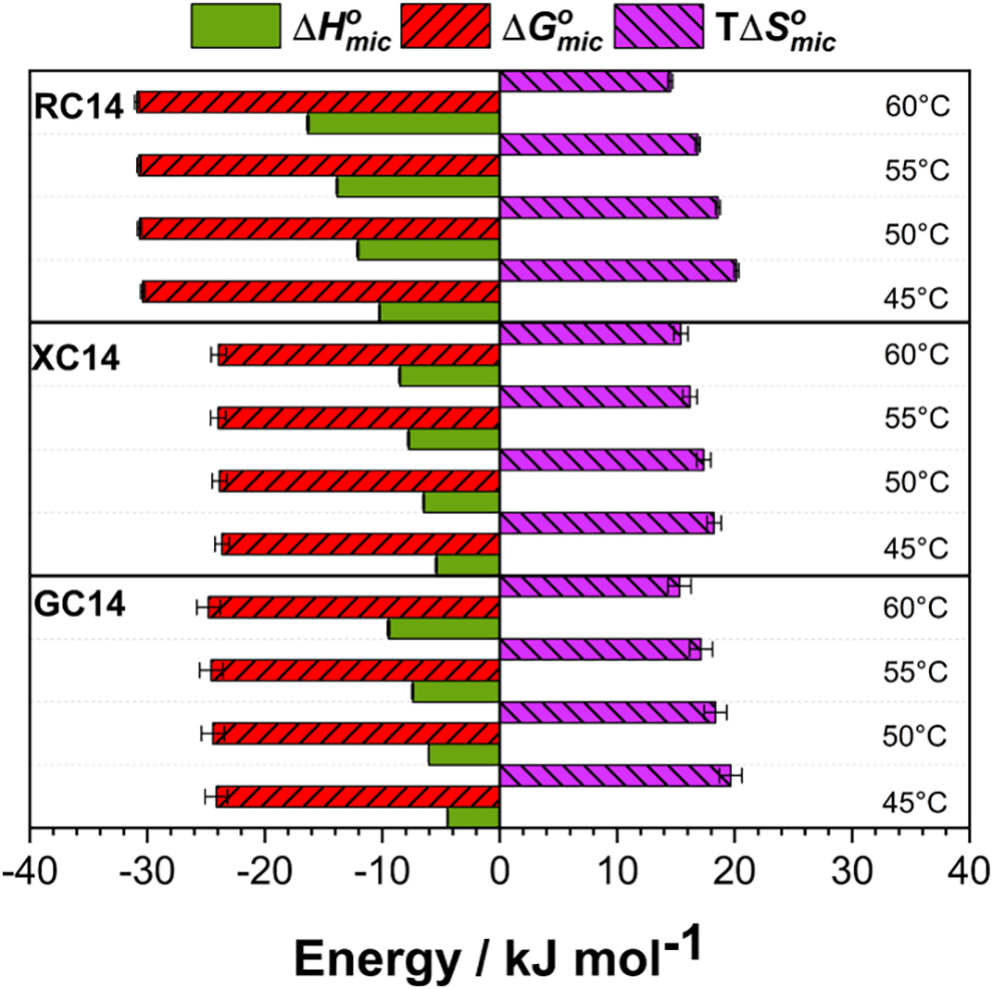
Thermodynamic parameters for the micellization for GC14, XC14, and RC14 glycolipids, in pH 7.4 at different temperatures. The error bars represent the uncertainty calculated through the propagation of deviations.

The CMC values reported in Table 3 follow the same trend observed in the surface tension data. Although the CMC values obtained from surface tension measurements and calorimetry differ quantitatively due to the distinct operational definitions of the CMC associated with each technique, both methods consistently capture the same relative trend among the glycolipids. To ensure accurate determination of the degree of ionization (α), measurements were conducted in pure water rather than in phosphate buffer, owing to the high conductivity of the latter. Under buffered conditions, the elevated ionic strength and background conductivity can obscure the contribution of the glycolipid, thereby compromising the precision of the α measurements. The α values obtained in water are expected to be higher than those measured in buffer, leading to an overestimation of $\Delta G_{mic}^{o}$ (Equation 10). Nevertheless, XC14 is likely to remain more dissociated than RC14 and GC14 even in buffer, thus preserving the validity of the comparison of the thermodynamic functions of micellization among the different glycolipids.

For all investigated systems, $\Delta G_{mic}^{o}$ is negative, indicating that micelles, with ellipsoidal morphology, are thermodynamically favored over free glycolipid monomers at equilibrium. This parameter remains essentially unchanged with increasing temperature and highlights greater thermodynamic stability for the glycolipid with the lowest hydrophilicity, RC14. To rationalize the behavior of $\Delta G_{mic}^{o}$ , it is necessary to analyze the other thermodynamic functions, $\Delta H_{mic}^{o}$ and $T\Delta S_{mic}^{o}$ .

Micellization was both enthalpically and entropically favorable under all experimental conditions, as evidenced by negative $\Delta H_{mic}^{o}$ and positive $T\Delta S_{mic}^{o}$ values. With increasing temperature, the enthalpic favorability becomes more pronounced (i.e., $\Delta H_{\mathrm{mic}}^{o}$ becomes more negative), while the entropic contribution gradually decreases. This opposing trend highlights a clear enthalpy–entropy correlation effect (Figure S7 in Section S5 of the Supporting Information). Such behavior is intrinsically linked to changes in water structure and dynamics, both in the bulk and at the micellar surface, where solvation becomes less effective at elevated temperatures.^50–52^

The enthalpic and entropic aspects of micellization for ionic glycolipids are attributed to two primary factors: *i*) dispersive interactions among alkyl chains in the aggregated state, as well as electrostatic interactions between headgroups and counterions, which offset the enthalpic contributions from monomer desolvation and headgroup–headgroup repulsion within the micelle;^53,54^ and *ii*) an increase in entropy, mainly due to the release of structured water molecules surrounding monomers upon their desolvation.^54^

To highlight the effect of structural differences among the sugar headgroups, we consider the magnitudes of $\Delta H_{mic}^{o}$ and $T\Delta S_{mic}^{o}$ values at 45 °C (Table 3). Enthalpic favorability follows the order RC14 > XC14 > GC14, while entropic favorability follows RC14 > GC14 > XC14. These results indicate that RC14, the least hydrophilic glycolipid, shows the greatest overall thermodynamic driving force, with favorable contributions from both $\Delta H_{mic}^{o}$ and $T\Delta S_{mic}^{o}$ . This suggests that the hydroxyl on the sugar headgroups contribute to surfactant solubility (as reflected in the CMC values), but also in interactions upon micellization that appear as enthalpic and entropic contributions. The fact that entropic contributions do not vary so much within the series may suggest that these hydroxyl groups are not substantially desolvated upon micellization.

On the other hand, the C5 substituent of the six-membered sugar ring plays a central role in modulating micellization, including its energetics, in agreement with our observations from surface tension and scattering experiments. In RC14, the C5 methyl group undergoes desolvation upon micellization, promoting exothermic dispersive interactions at the micelle core–corona interface and providing an additional entropic gain by removing a hydrophobic moiety from water. The enthalpic difference observed between RC14 and the other glycolipids is close to the reported contribution for desolvation of one −CH_3_ group.^55^ Although XC14 and GC14 display similar $\Delta H_{mic}^{o}$ values, GC14 shows a larger entropic gain, likely associated with partial desolvation of the −CH_2_OH group. However, its enthalpic contribution is less favorable, possibly due to conformational rearrangements of the headgroup upon micelle formation for packing, as also supported by structural analyses derived from SAXS measurements.

Earlier studies have shown that even subtle structural changes in sugars, particularly at a single carbon, can strongly alter their interactions.^56,57^ Thoden et al. (2002)^56^ demonstrated that the configuration of the one carbon is critical: in some sugars, a −CH_2_OH substituent at this position enables more hydrogen bonding, strengthening sugar–protein interactions. In the current context, xylose (a pentose) does not have the same arrangement as rhamnose, although a hexose like galactose, carries a −CH_3_ group instead of a hydroxyl, making it the most hydrophobic sugar in the present study. By contrast, galactose forms more hydrogen bonds, promoting interfacial dehydration and the development of a more compact structure.^57^ Subtle modifications, such as replacing a hydroxyl group with a methyl group, alter the balance between hydrogen bonding and hydrophobic interactions, thereby affecting micelle structure and stability and the overall thermodynamic profile of micellization. These findings highlight how energetic and structural factors may explain why some specifically sugar derivatives exert stronger biological effects and display distinct energetic behavior in glycolipid self-assembly.

## 4 Conclusions

In this work, we investigated the solubility, interfacial behavior, aggregate structure, and aggregation energetics of anionic bioinspired glycolipids with rhamnose, xylose, or galactose headgroups over a range of temperatures. Surface tension measurements revealed a direct correlation between their critical micelle concentrations (CMC) and the number of hydroxyl groups, following the order RC14 < XC14 < GC14. Although all glycolipids exhibited comparable surface activity, their surface molecular area (*a*_0_) values indicated deviations from ideal spherical micelle packing, which were further supported by light and X-ray scattering analyses that reveal formation of ellipsoidal aggregates.

Isothermal titration calorimetry (ITC) measurements provided further insight into the micellization process, indicating that while hydroxyl groups strongly influence solubility and, therefore, the CMC, they primarily affect the thermodynamic parameters of micellization, with a more pronounced impact on $\Delta H_{\mathrm{mic}}^{o}$ and $\Delta G_{\mathrm{mic}}^{o}$ , while $T\Delta S_{\mathrm{mic}}^{o}$ remains essentially unchanged. Instead, the dominant contribution to micellization energetics arises from the substituent at the C5 position of the sugar ring. The less hydrophilic RC14, bearing a methyl group at this position, exhibited the most favorable enthalpic and entropic contributions.

Overall, these results provide new insights into the self-assembly of ionic glycolipids, underscoring the role of headgroup chemistry in tuning the thermodynamic, structural, and interfacial properties of micellar ionic glycolipid system.

## Supplementary Material



## Notes

Section S1: determination of the acid constant; methods and results for characterization of protonation behavior of the compounds; Section S2: determination of critical micelle concentration (CMC); degree of ionization (α) from electrical conductivity measurements; quantification of micellization onset; extent of dissociation in solution; Section S3: dynamic light scattering (DLS) data; hydrodynamic size distribution; aggregation behavior of micelles in solution; Section S4: estimation of structural parameters; aggregation numbers; calculated micelle size; aggregation number; related geometric properties derived from experimental data; Section S5: isothermal titration calorimetry (ITC) results; thermodynamic parameters; enthalpy changes associated with micelle formation; interactions in solution; Section S6: compilation of supplementary references; support for methodologies; analyses; theoretical interpretations (PDF)
