# Supplementary material for: Influence of Sugar Headgroup on the Self-Assembly of Bioinspired Anionic Glycolipids
Source: Langmuir. 2026 Feb 3;42(6):4549–58. doi: 10.1021/acs.langmuir.5c05353 (PMC12922182; doi:10.1021/acs.langmuir.5c05353)
Supplement: Supplementary file 1 [file la5c05353_si_001.pdf]

**Supporting Information for:**

# Influence of Sugar Headgroup on the Self-Assembly of Bioinspired Anionic Glycolipids

Giuliana Valentini, Álvaro Javier Patiño-Agudelo, Paulo Ricardo de Abreu Furtado Garcia, Watson Loh*

*Institute of Chemistry, State University of Campinas (UNICAMP), P.O. Box 6154, 13083-970 Campinas, SP, Brazil.*

**Corresponding author:* wloh@unicamp.br

# Table of Contents

**Section S1.** Acid constant determination…………………………………………………(3)

**Section S2.** Determination of CMC and α Using Electrical Conductivity ……….……(5)

**Section S3.** Dynamic Light Scattering (DLS) …………………………………………(7)

**Section S4.** Structural Parameters and Aggregation Number Estimations……………..(8)

**Section S5.** Isothermal Titration Calorimetry (ITC) …………………………………..(9)

**Section S6.** Supplementary References………………………..……………...……(13)

## Section S1. Acid constant determination

### *1.1 Description of $pK_a$ measurements*

The $pK_a$ was determined by inverse potentiometric titration due to the low solubility of the protonated species above the CMC in water.[1] In a thermostated titration cell (60°C) containing 50.00 mL of the glycolipid solution, a known excess of 0.1 mol $L^{-1}$ NaOH was added to ensure complete conversion to the carboxylate salt and full solubilization (initial pH ≈ 12). Under magnetic stirring, and with pH measurements performed using a calibrated pH meter (Titrino Plus, model 848, Metrohm, Herisau, Switzerland), the excess base was titrated with 0.1 mol $L^{-1}$ HCl in small increments near the equivalence point, recording the pH after stabilization. The pKa was determined as the pH corresponding to the condition [COOH] = [$COO^-$], calculated from the equivalence volume obtained by the first derivative of the titration curve.

### *1.2 Results of $pK_a$ measurements*

Figure S1 shows potentiometric titration curves and their first derivatives for three systems (GC14, XC14, and RC14). In Figure S1a, the pH variation as a function of the added HCl volume is presented. All three sugar-based surfactants start at alkaline values around pH 12. As the acid is added, the curves display two plateaus followed by sharp decreases in pH, which correspond to protonation events of the systems. The first event corresponds to the neutralization of the NaOH excess (around pH 7), while the second event is associated with the neutralization of the carboxylic acid groups (around pH 4–5).[2] This behavior is more clearly described in Figure S1b, which shows the first derivative of the titration curves ($\left(\frac{\partial pH}{\partial V}\right)_T$ as a function of pH). The observed minimum corresponds to the $pK_a$ values of the systems, as summarized in Table S1.

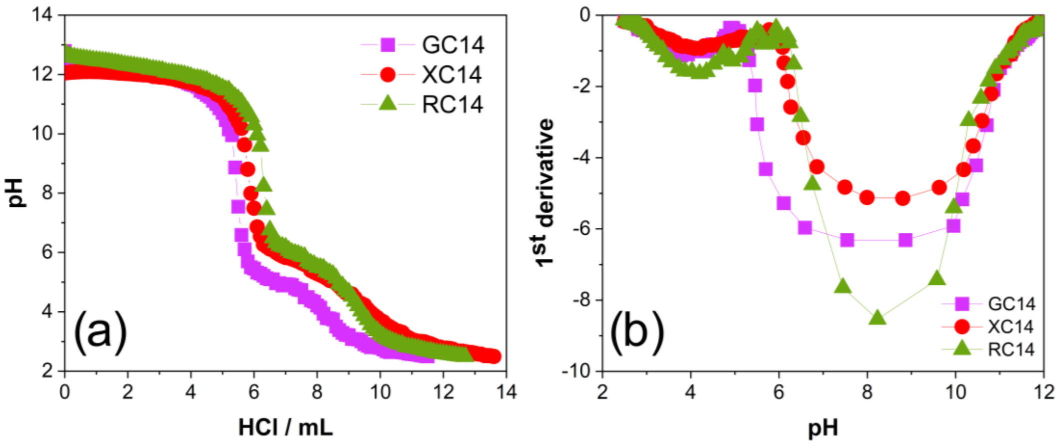


**Figure S1.** Potentiometric titration curves of glycolipids with C14 alkyl chains at 60°C. (a) pH as a function of the added HCl volume for GC14, XC14, and RC14. (b) First derivative $\left(\frac{\partial pH}{\partial V}\right)_T$ of the titration curves plotted against pH, highlighting the apparent $pK_a$ values for each system.

**Table S1.** $pK_a$ for RC14, XC14 and GC14.

| **Sample** | **$pK_a$** |
|---|---|
| **RC14** | 4.3±0.0 |
| **XC14** | 4.2±0.1 |
| **GC14** | 4.1±0.2 |

## Section S2. Determination of CMC and α Using Electrical Conductivity

### *2.1 Measurements of electrical conductivity*

Figure S2 shows the variation of conductivity with the concentration of sugar-based surfactants in pure water at different temperatures. Each curve was measured in triplicate. The temperatures were selected based on the Krafft point, representing the operational temperature range of the system.

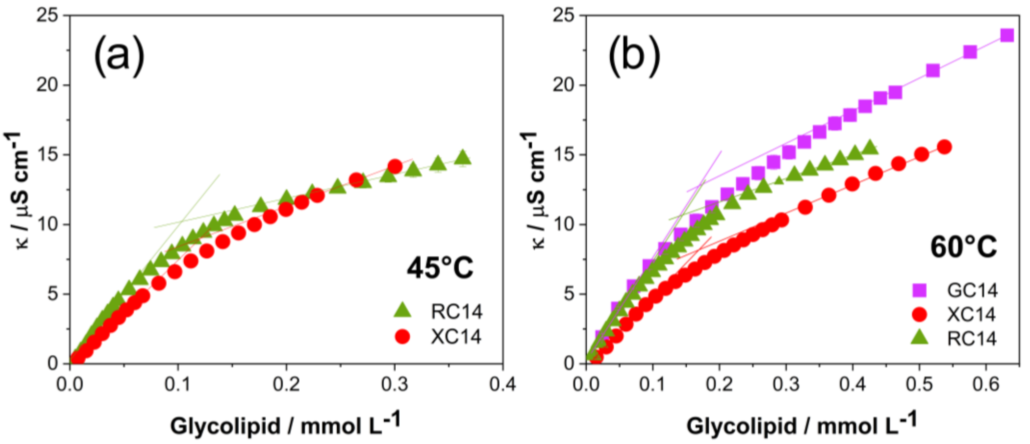


**Figure S2.** Conductivity as a function of sugar-based surfactant concentration in water. (a) XC14 and RC14 at 45°C; (b) GC14, XC14 and RC14 at 60°C. GC14 could not be measured at 45°C due to its high Krafft temperature in water (~59 °C), which is higher than in phosphate buffer (~41°C). The initial and final pH values of the samples at 60 °C were: GC14 (initial pH: 7.09, final pH: 4.8); XC14 (initial pH: 7.1, final pH: 5.5); RC14 (initial pH: 7.1, final pH: 5.1).

The conductivity curves did not display the typical two linear regions separated by a distinct break point, as commonly observed for ionic surfactants when determining the CMC. Instead, the transition was more gradual, resembling an exponential behavior, with the entire curve accompanied by pH variations across the concentration range, suggesting that $H^+$ ions, in addition to $Na^+$, are adsorbed onto the micellar surface.[3] The micellar degree of dissociation (α) and the CMC were determined by performing linear fits to the initial and final regions of the curve, using extrapolation and interpolation to obtain these parameters, and the results are summarized in Table S2. Under buffered conditions, the elevated ionic strength and background conductivity can obscure the contribution of the glycolipid, thereby compromising the precision of the α measurements. Therefore, the α values obtained in pure water were assumed to be a reasonable approximation for determining the thermodynamic potential of micellization under all studied conditions, since they are expected to be ≤ than those in buffered solutions.[3] As shown in Table S2, α values were not affected by temperature.

Statistical analysis[4] presented in the Table S2 showed no significant variance within replicates ($F = 1.0 < 19$, a 95% confidence level). Because α for GC14 could not be measured at 45 °C due to its Krafft point, and RC14 and XC14 exhibited essentially identical α values at 45 °C and 60 °C, the α value determined at 60 °C was assumed to be constant and was therefore used for all glycolipids in subsequent calculations.

**Table S2.** The comparison of CMC (in mmol $L^{-1}$) and α in water to GC14, XC14 and RC14.

| Sample | GC14 | XC14 | RC14 |
|---|---|---|---|
| **CMC (60°C)** | 0.20 ± 0.03 | 0.16 ± 0.02 | 0.14 ± 0.02 |
| **CMC (45°C)** | - | 0.11 ± 0.01 | 0.10 ± 0.01 |
| **α 60°C (n=3)** | 0.24 ± 0.07 | 0.45 ± 0.04 | 0.23 ± 0.02 |
| **α 45°C (n=3)** | - | 0.41 ± 0.04 | 0.19 ± 0.02 |
| **α t-calc** | - | 1.23 | 2.45 |
| **α t-crit** | - | 2.776 | 2.776 |
| **α significant difference** | - | No | No |

The α means between paired samples was performed using the classical Student's *t*-test, assuming equal variances ($F_{calculated} < F_{critical}$). For each pair, the mean values and standard deviations (n = 3) are reported along with the calculated sample variances.

**Section S3. Dynamic Light Scattering (DLS)**

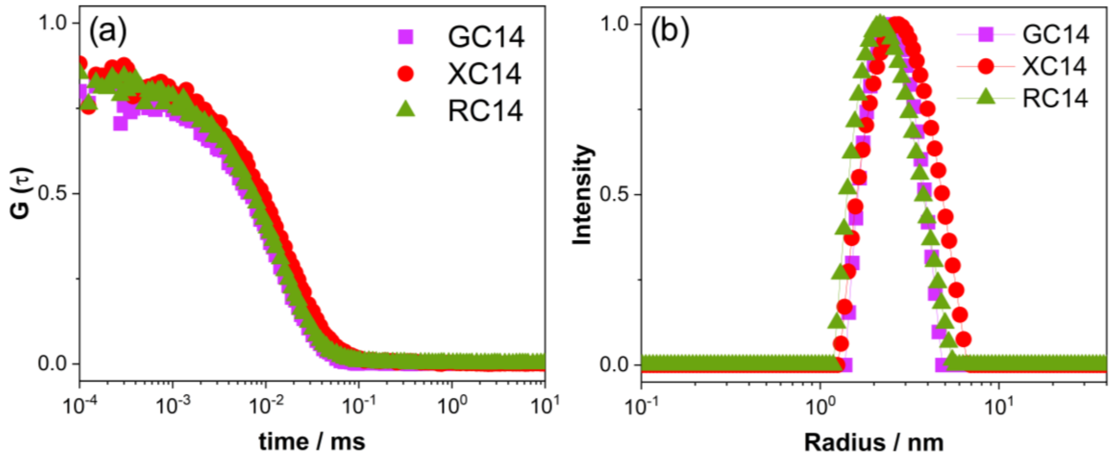


**Figure S3. (a)** Intensity-time autocorrelation and **(b)** Distribution of hydrodynamic radii for GC14, XC14 and RC14, at 45°C.

## Section S4. Structural Parameters and Aggregation Number Estimates

*4.1 Description of structural parameter measurements*

The length of the hydrocarbon chain ($l_0$) was estimated using Equation S1, and the volume of the hydrophobic chain ($V_{chain}$) was calculated based on the Tanford model (Equation S2).[5] The ellipsoidal core volume ($V_{core}$) was calculated using Equation S3 and the fitting parameters, where the XY axes (a = b = R) and the Z axis are defined as c = R × ϵ.[6,7] For the core + shell volume, the shell thickness ($th$) is applied uniformly to all axes, and the calculation follows Equation S4. The aggregation number ($N_{agg}$) was then estimated as the ratio between the core volume and the volume of a single hydrophobic chain, both without (Equation S5) and with consideration of the shell (Equation S6).

$$l_0(nm) = 0.154 + 0.1265(n) \tag{S1}$$

$$V_{chain}(nm^3) = 0.0274 + 0.0269(n) \tag{S2}$$

$$V_{core} = \frac{4}{3}\pi\epsilon R^3 \tag{S3}$$

$$V_{core+shell} = \frac{4}{3}\pi\epsilon[(R + th)^3] \tag{S4}$$

$$N_{agg\ core} = \frac{V_{core}}{V_{chain}} \tag{S5}$$

$$N_{agg\ core+shell} = \frac{V_{core+shell}}{V_{chain}} \tag{S6}$$

**Section S5. Isothermal Titration Calorimetry (ITC)**

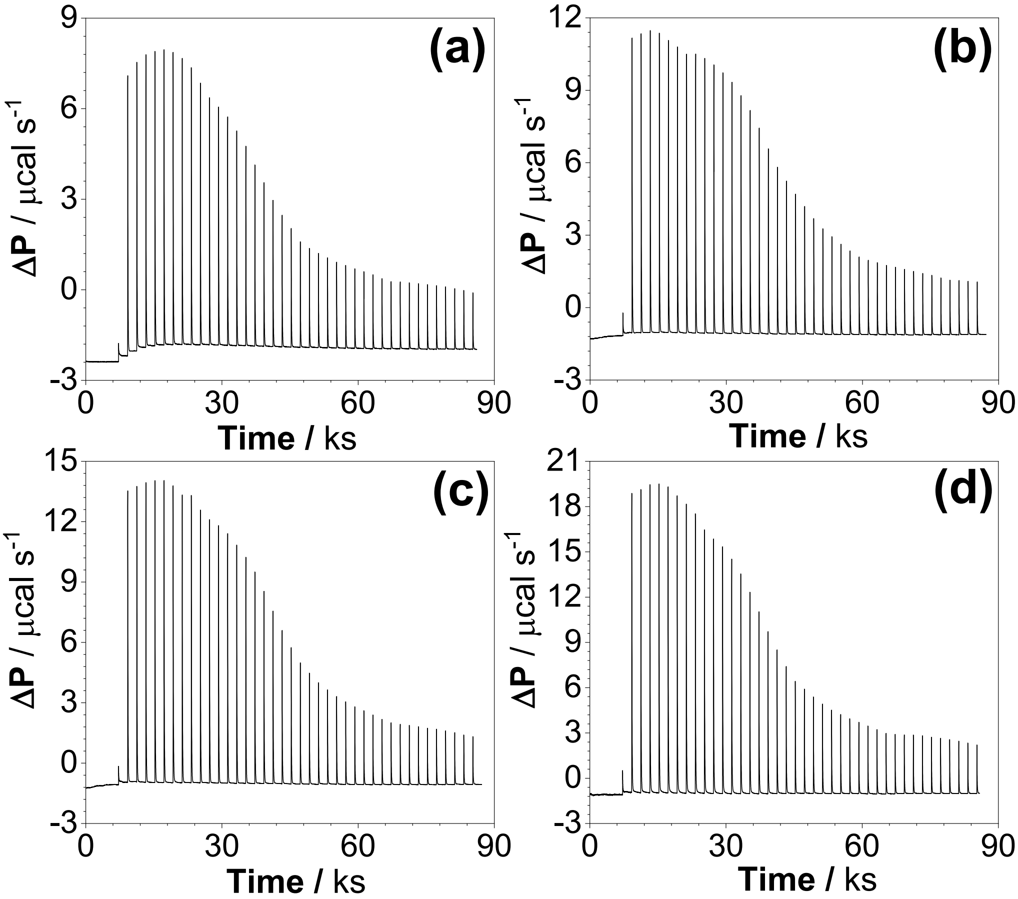


**Figure S4.** Thermal power as a function of time plotted for sequential 5 µL injections of a concentrated solution of GC14 into 1.44 mL of solvent at: **(a)** 45 °C, **(b)** 50 °C, **(c)** 55 °C, and **(d)** 60 °C.

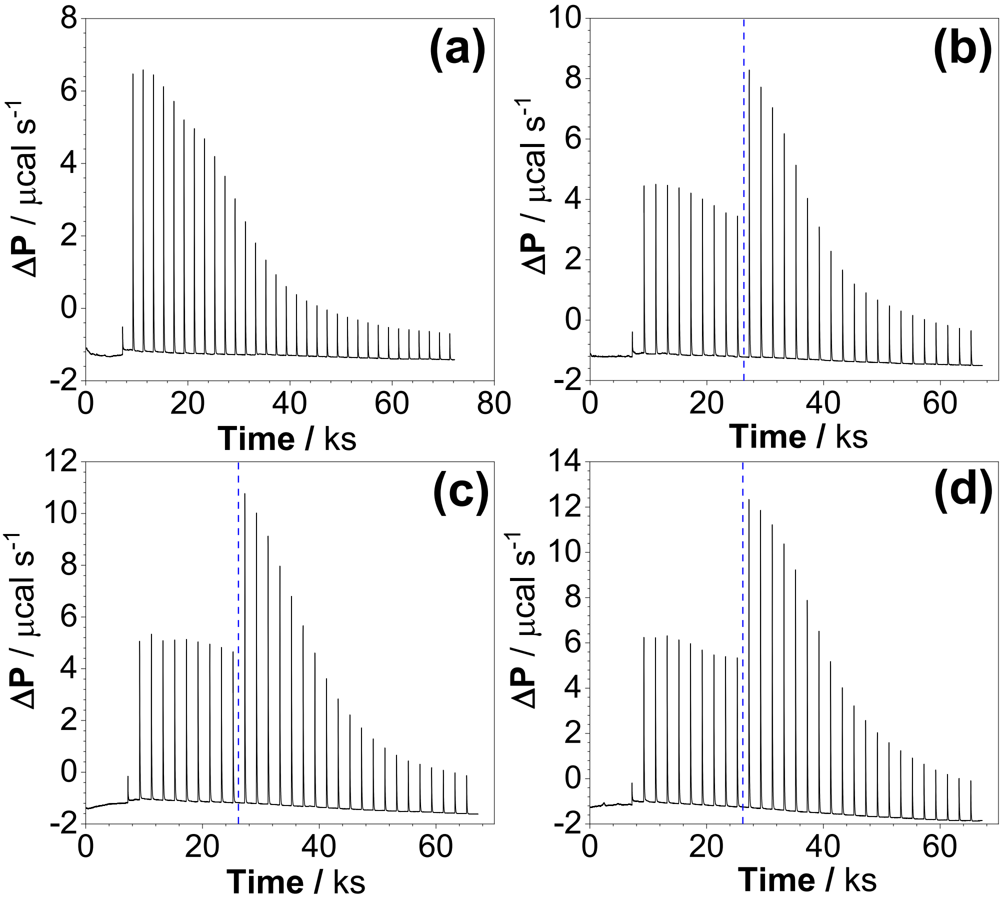


**Figure S5.** Thermal power as a function of time for sequential injections of a concentrated XC14 solution into 1.44 mL of solvent at **(a)** 45 °C, **(b)** 50 °C, **(c)** 55 °C, and **(d)** 60 °C. In **(a)**, all injections were 5 μL. In **(b)**, **(c)**, and **(d)**, 5 μL injections were made before the vertical blue dashed line, followed by 10 μL injections. The change in injection volume was necessary due to instrument limitations during a unique experiment, as the thermal power generated by volumes above 5 μL at higher temperatures exceeded the detection range.

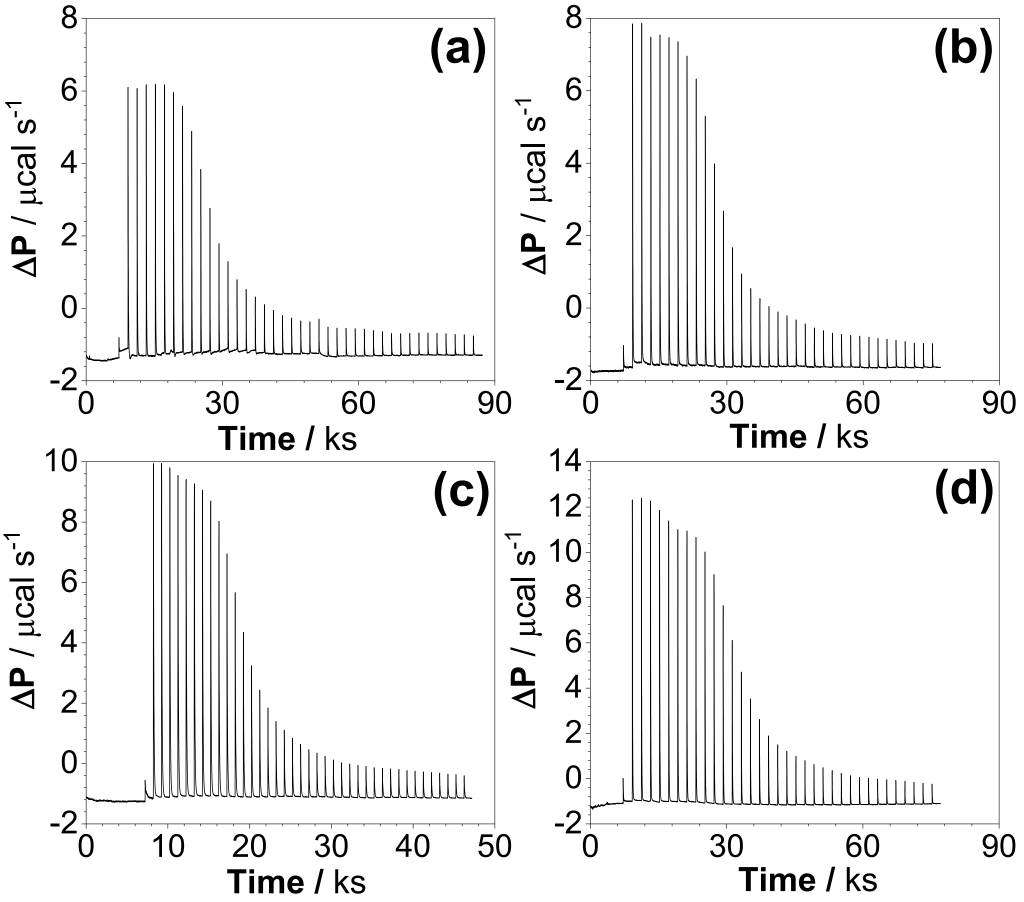


**Figure S6.** Thermal power as a function of time plotted for sequential 5 µL injections of a concentrated solution of RC14 into 1.44 mL of solvent at: **(a)** 45 °C, **(b)** 50 °C, **(c)** 55 °C, and **(d)** 60 °C.

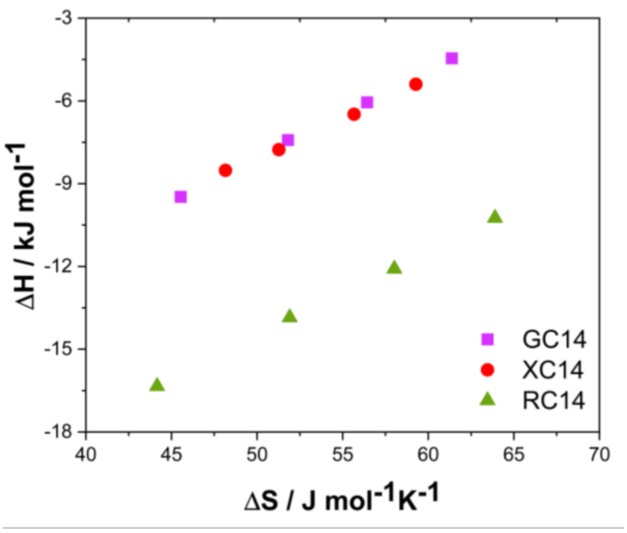


**Figure S7.** Enthalpy–entropy correlation plot of GC14, XC14, and RC14 micellization.

## Section 6. Supplementary References


(1) Drzymala, J. Potentiometric Titration of Sodium Oleate in Dilute Aqueous Solutions. *J. Colloid Interface Sci.* **1985**, *107* (2), 442–445.

(2) Lebrón-Paler, A.; Pemberton, J. E.; Becker, B. A.; Otto, W. H.; Larive, C. K.; Maier, R. M. Determination of the Acid Dissociation Constant of the Biosurfactant Monorhamnolipid in Aqueous Solution by Potentiometric and Spectroscopic Methods. *Anal. Chem.* **2006**, *78* (22), 7649–7658.

(3) Ikeda, S.; Tsunoda, M.-A.; Maeda, H. The Effects of Ionization on Micelle Size of Dimethyldodecylamine Oxide. *J. Colloid Interface Sci.* **1979**, *70* (3), 448–455.

(4) Skoog, D. A.; West, D. M.; Holler, F. J.; Crouch, S. R. *Fundamentals of Analytical Chemistry*, 9th ed.; Brooks/Cole: Boston, 2013.

(5) Tanford, C. *The Hydrophobic Effect: Formation of Micelles and Biological Membranes*, 2nd Edition.; Wiley-Interscience Publication, 1980.

(6) Mortensen, H. G.; Madsen, J. K.; Andersen, K. K.; Vosegaard, T.; Deen, G. R.; Otzen, D. E.; Pedersen, J. S. Myoglobin and α-Lactalbumin Form Smaller Complexes with the Biosurfactant Rhamnolipid Than with SDS. *Biophys. J.* **2017**, *113* (12), 2621–2633.

(7) López Hernández, M.; Otzen, D. E.; Pedersen, J. S. Investigating the Interactions between an Industrial Lipase and Anionic (Bio)Surfactants. *J. Colloid Interface Sci.* **2025**, *679*, 294–306.
